# Transcriptome Profiles of the Liver in Two Cold-Exposed Sheep Breeds Revealed Different Mechanisms and Candidate Genes for Thermogenesis

**DOI:** 10.1155/2021/5510297

**Published:** 2021-08-10

**Authors:** Dan Jiao, Kaixi Ji, Wenqiang Wang, Hu Liu, Jianwei Zhou, A. Allan Degen, Yunsheng Zhang, Ping Zhou, Guo Yang

**Affiliations:** ^1^Northwest Institute of Ecological Environment and Resources, Chinese Academy of Science, Lanzhou 730000, China; ^2^University of Chinese Academy of Sciences, Beijing 100049, China; ^3^Key Laboratory of Stress Physiology and Ecology, Northwest Institute of Ecological Environment and Resources, Chinese Academy of Science, Lanzhou 730000, Gansu, China; ^4^School of Life Sciences, Lanzhou University, Lanzhou 730020, China; ^5^Desert Animal Adaptations and Husbandry, Wyler Department of Dryland Agriculture, Blaustein Institutes for Desert Research, Ben-Gurion University of Negev, Beer Sheva 8410500, Israel; ^6^Institute of Animal Husbandry, Xinjiang Academy of Animal Science, Urumqi 830000, Xinjiang, China; ^7^State Key Laboratory of Sheep Genetic Improvement and Healthy Production, Xinjiang Academy of Agricultural and Reclamation Science, Shihezi 832000, China

## Abstract

Cold-induced thermogenesis plays an important role in the survival of lambs exposed to low air temperatures. The liver produces and mediates heat production in mammals; however, to date, little is known about the role of liver genes in cold-induced thermogenesis in lambs. In this study, the difference in the liver transcriptome between Altay and Hu ewe lambs was compared. Because of different backgrounds of the two breeds, we hypothesized that the transcriptome profiles of the liver would differ between breeds when exposed to cold. Cold-exposed Altay lambs activated 8 candidate genes (*ACTA1*, *MYH1*, *MYH2*, *MYL1*, *MYL2*, *TNNC1*, *TNNC2*, and *TNNT3*) involved in muscle shivering thermogenesis; 3 candidate genes (*ATP2A1*, *SLN*, and *CKM*) involved in muscle nonshivering thermogenesis related to the Ca^2+^ signal and creatine cycle; and 6 candidate genes (*PFKM*, *ALDOC*, *PGAM2*, *ENO2*, *ENO3*, and *ENO4*) involved in enhancing liver metabolism. In contrast, the liver may not act as the main tissue for thermogenesis in cold-exposed Hu lambs. We concluded that Altay lambs rely on liver-mediated shivering and nonshivering thermogenesis by muscle tissue to a greater extent than Hu lambs. Results from this study could provide a theoretical foundation for the breeding and production of cold-resistant lambs.

## 1. Introduction

Sheep are thermostable animals and rely on thermogenesis to maintain a relatively constant body temperature; however, exposure to extreme cold can cause hypothermia and, ultimately, high mortality in lambs. Prolonged exposure to cold results in the activation of hormonal and metabolic responses in different tissues [1], including nonshivering thermogenesis (NST) in brown adipose tissue (BAT) [2]. Adrenergic activated NST involves uncoupling protein 1 (*UCP1*) that facilitates proton leakage across the inner mitochondrial membrane, leading to futile cycling of protons and heat generation [3]. It has been estimated that NST contributes approximately 12–20 percent of the increase in total daily heat production, while NST in BAT contributes approximately 2–17 percent of the increase [4], which suggests that other tissues are also involved in the process. Recently, the liver has been reported to play an important role in providing “fuel” for NST by BAT, at least in cold-exposed mice [5].

The liver produces approximately 10–13 percent of the basal heat production of mammals and has been closely linked with thermogenesis [6]. In rats, body temperature and heat production from the liver decreased when NST was inhibited [7]. Bond and Ntambi [8] reported that the liver was related to an increase in adipose tissue and the synthesis and transport of monounsaturated fatty acids in UCP1-deficient mice. Some liver-derived factors, including fibroblast growth factor 21 (*FGF21*), have been reported to be important in the regulation of thermogenesis during acute cold exposure in mice [9]. However, the genetic mechanisms of the liver during cold exposure in lambs are still unclear. To fill this gap, at least in part, we examined the changes in the expression of liver-derived genes in two sheep breeds exposed to cold. Altay sheep are indigenous in the Altay Prefecture of the northern Xinjiang province and are well adapted to the harsh conditions. This region is extremely cold in winter with an average January temperature of −16.3°C (https://Climate-Data.org) and with a long period of winter snow cover. In contrast, Hu sheep are typically raised in the subtropical climate zone of China, a region characterized by warm, humid conditions. In a companion study on the Altay and Hu sheep, maintenance energy requirements were higher for Hu than for Altay sheep at −5°C, and this temperature was below the thermoneutral zone for Hu sheep but within the thermoneutral zone for Altay sheep [10]. Because of different backgrounds and responses to cold exposure between the two breeds, we hypothesized that the transcriptome profiles of their livers would differ when exposed to cold. Results of this study provide (1) a better understanding of the role of the liver in thermogenic mechanisms employed by lambs and (2) a research base for improving the cold tolerance in sheep.

## 2. Materials and Methods

### 2.1. Animals and Ethics

The study design and all procedures on sheep were approved by the Academic Committee of the Northwestern Institute of Eco-Environment Resources, Chinese Academy of Sciences (protocol no. CAS201810082). Twelve Altay (A) and 12 Hu (H) ewe lambs, all six months of age and of similar body weight (29.3 ± 2.47 kg), were purchased from a feedlot in the Altay region and were raised at the Gansu Gaolan Field Scientific Observation and Research Station for Agricultural Ecosystem (36°14′16 N, 103°47′59E). Wool length did not differ between the two breeds and averaged 8.87 ± 2.15 cm. The lambs were fed alfalfa pellets *ad libitum*, with free access to water, prior to and during the study. They were maintained in individual metabolic cages (1.5 m × 1.0 m), and each breed was divided into two groups: chronic cold-exposed (6 A^c^ and 6 H^c^), which were maintained in a room at a constant temperature of −5°C for 25 days, after a gradual decrease of 2.5°C/day over 10 days, and control (6 A^w^ and 6 H^w^), which were maintained in a room at a constant temperature of 20°C throughout this period. The temperature and humidity of the cold room were −5°C ± 0.03 and 88% ± 6.5, respectively, and of the thermoneutral room were 20°C ± 0.42 and 87.5% ± 9.9, respectively. The temperature humidity indices (THI), following Tucker et al. [11], were 26 ± 0.18 and 63 ± 0.62, respectively. All lambs were slaughtered after 12 h of fasting in the morning after 25 days of temperature exposure, and their livers were excised and immediately frozen in liquid nitrogen and then stored at −80°C.

### 2.2. RNA Extraction, Library Construction, and Sequencing

Total RNA was extracted from the liver of 19 lambs (A^c^ (*n* = 5), A^w^ (*n* = 4), H^c^ (*n* = 6), and H^w^ (*n* = 4)) using TRIzol reagent (Invitrogen, Carlsbad, CA, USA) according to the manufacturer's protocol. The RNA quality and integrity number (RIN) were measured using Agilent 2100 (Agilent Technologies, Santa Clara, CA, USA). The concentration and purity of the RNA samples were tested through the threshold filter of RIN >7.0 and 28S/18S rRNA ratio >1.0 to ensure that RNA quality meets sequencing standards. The RNA samples from individual lambs in every group (independent biological replicates) were not pooled in order to exclude samples with poor biological duplication to ensure the reliability of all sequencing results and subsequent analyses. Consequently, of 24 liver samples (12 Hu and 12 Altay lambs) sequenced, 19 were used for analysis. Poly A messenger RNA (mRNA) was isolated from total RNA by an oligo dT extraction kit (NEBnext Poly(A) mRNA Magnetic Isolation Module, NEB, USA) and then fragmented using divalent cations under elevated temperature. First-strand cDNA was synthesized from fragmented mRNA using random oligonucleotide primers and reverse transcriptase (SuperScript II Reverse Transcriptase, Invitrogen, Carlsbad, CA, USA) and second strand from DNA polymerase I and RNase H treatments. The cDNA fragments' production had a single “A” nucleotide base added, followed by ligation of an adapter. The products were purified by AMPure XP beads and then dissolved in EB solution and enriched with PCR amplification to create the final cDNA library. The overall quality of the PCR product was validated by the Agilent Technologies 2100 bioanalyzer. The double-stranded PCR products were heated, denatured, and circularized by the splint oligo sequence to obtain the final library. The cDNA fragments in the library were sequenced using a BGISEQ-500 platform (Beijing Genomics Institute (BGI), Beijing, China) for producing raw reads.

### 2.3. RNA-Seq Data Analysis

The clean reads were filtered out based on the raw reads, using quality control software SOAPnuke (BGI), which were obtained by removing low-quality reads (more than 20 percent of bases in the total reads had quality scores lower than 15), adaptor reads (reads with joint contamination), and unknown base *N* content (reads which contain more than 5 percent undetermined base information). The reads were aligned and annotated to the reference genome of *Ovis aries* (Oar_rambouillet_v1.0; https://www.ncbi.nlm.nih.gov/assembly/GCF_002742125.1) using the HISAT alignment tool (Centre for Computational Biology, Johns Hopkins University, MD, USA). HISAT is based on the Burrows–Wheeler transform and Ferragina–Manzini (FM) indexing methods [12]. We used Bowtie 2 for calculating the gene alignment rate [13] (Johns Hopkins University, MD, USA) and then calculated gene expression levels with RSEM (version 1.2.12, University of Wisconsin–Madison, USA), a software package for estimating gene and isoform expression levels from RNA-Seq data [14]. The gene expression levels were standardized by reads per kilobase per million (FPKM) mapped reads. The constrained principal coordinate analysis (cPCoA) was employed to visualize classical multidimensional scaling of Bray–Curtis distance matrices by using functions capscale and anova.cca of vegan package in R (version 4.0, Lucent Technologies, AZ, USA), and the *P* value was calculated by permutation tests [15, 16].

### 2.4. Differentially Expressed Gene Analysis

We compared differential gene expressions in the liver between breeds and between air temperatures using pairwise comparisons, as described by Wang et al. [17]. Differentially expressed genes (DEGs) were filtered by DESeq2 software and as fold changes (FC, |log2 FC| > 1) and *q* value (*q* < 0.05). The *q* value is based on the multiple hypothesis testing on the *P* value. Fold change (FC) was calculated as follows:

(1) $$\begin{array}{c} \text{FC}=\frac{\text{avg FPKM}\left(-5^{{}^{\circ}}\text{C}\right)}{\text{avg FPKM}\left(20^{{}^{\circ}}\text{C}\right)}. \end{array}$$

DEGs meeting the above screening criteria were carried out by subsequent clustering analysis.

### 2.5. Function Enrichment and Analyses

Gene Ontology (GO) [18] and Kyoto Encyclopedia of Genes and Genomes (KEGG) [19] enrichment analyses were based on DEGs. GO terms were enriched by phyper functions in R software (version 4.0, Lucent Technologies, AZ, USA), and based on the GO annotation results, DEGs were mapped to the GO terms in the database (https://www.geneontology.org/). The KEGG pathway enrichment was used to identify genes and the metabolic pathways involved in the DEGs (https://www.kegg.jp/kegg/pathway.html/), and this method was consistent with the GO enrichment. In order to further study the effect of cold exposure on gene transcription regulation in sheep, we enriched the up- and downregulated genes into GO terms and KEGG pathways, respectively. A level of *q* value < 0.05 was accepted as a significant difference between means.

### 2.6. RNA-Seq Validation by Quantitative Real-Time PCR

To validate the gene expression differences that had been identified by the RNA-seq analysis, four candidate genes, namely, apolipoprotein D (*APOD*), apolipoprotein A4 (*APOA4*), lipin (*LPIN*), and trefoil factor 2 (*TFF2*), were identified using an RT-qPCR approach. These four genes were significantly regulated in lambs by cold exposure, and their expression levels were high; they also play important roles in liver metabolism.

Total RNA samples were obtained from the liver of 19 lambs (A^c^ (*n* = 5), A^w^ (*n* = 4), H^c^ (*n* = 6), and H^w^ (*n* = 4)), which were used for transcriptome sequencing. The primers for RT-qPCR were designed using Oligo 7 (Wojciech Rychlik, USA) (Table 1), with *β*-actin as a reference gene to certify the relative level of expression. The RT-qPCR amplification of cDNA pools used a PrimeScript RT reagent kit (Takara) with gDNA Eraser (Takara) according to the manufacturer's instructions. The RT-qPCR reactions were performed on Mx3000P and Mx3005P QPCR Systems (Stratagene, Agilent, Santa Clara, CA, USA). The qPCR reaction system was performed in a total volume of 20 *μ*L containing 2 *μ*L of cDNA, 0.8 *μ*L forward and reverse primers (10 *μ*M), 10 *μ*L TB Green^TM^ Premix Ex Taq II, 6 *μ*L RNase-free water, and 0.4 *μ*L ROX Reference Dye II (50×). The thermal profile for amplification was a two-step approach, which, after predegeneration, consisted of 15 s at 95°C in the first step and then 5 s at 95°C and 34 s at different Tm for 40 cycles in the second step. Changes in gene expressions were determined by the 2^−△△ct^ method [20]. Relative quantity between treatment and control groups was tested using *t*-test.

### 2.7. Statistical Analyses

Data are expressed as means ± SE, and all statistical analyses were performed by *t*-tests using SPSS software (SPSS version 17.0, Chicago, IL, USA).

## 3. Results

### 3.1. Sequencing and Mapping

To examine the global difference between breeds and treatments in the transcriptome sequences, constrained principal coordinate analysis (cPCoA) by Bray–Curtis distances was employed for every biological replicate of each tissue and treatment (Figure S1). It emerged that the 19 samples explained 16.4% of the variance of the total sequencing data, and the two breeds clustered well (*P*=0.43). The livers of –5°C and 20°C Hu lambs were distinct in CPCo 1 (explained 47.1% of 16.4% of the variance of the total sequencing data), whereas the liver tissues were distinct between breeds in CPCo 2 (explained 30.4% of 47.1% of the variance in CPCo 1). The results indicated that the transcriptome sequences in liver tissues separated the two breeds and separated the two temperature treatments in the Hu lambs.

The alignments for the genome and gene sequences were all made with Oar_rambouillet_v1.0. The sequencing and mapping data are summarized in Table S1. The results indicated that sequencing quality met the requirements for subsequent analysis.

### 3.2. Gene Annotation

**Figure 1:**
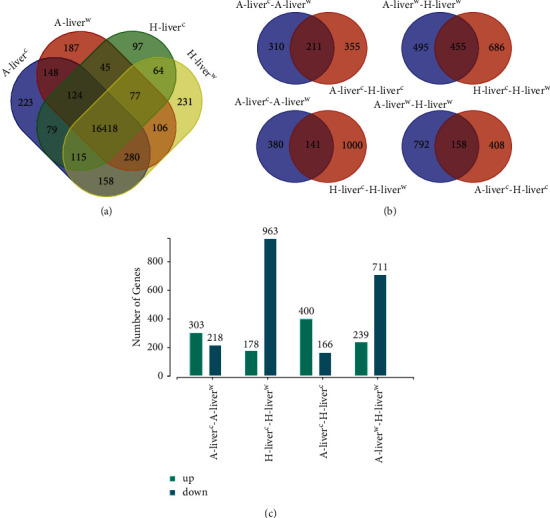
DEGs in the liver of cold-exposed Altay and Hu lambs. A-liver^c^-A-liver^w^: liver of −5°C Altay lambs (*n* = 5) compared to 20°C Altay lambs (*n* = 4); H-liver^c^-H-liver^w^: liver of −5°C Hu lambs (*n* = 6) compared to 20°C Hu lambs (*n* = 4); A-liver^c^-H-liver^c^: liver of −5°C Altay lambs (*n* = 5) compared to −5°C Hu lambs (*n* = 6); A-liver^w^-H-liver^w^: liver of 20°C Altay lambs (*n* = 4) compared to 20°C Hu lambs (*n* = 4). (a) DEGs among different treatment groups. (b) DEGs between differently compared groups. (c) The up- and downregulated DEGs between differently compared groups. The screen of DEGs is based on |log2 FC| > 1 and *q* value < 0.05. The green columns represent upregulated DEGs, and blue columns represent downregulated DEGs.

A total of 27,298 genes were annotated to the *Ovis aries* reference genome Oar_rambouillet_v1.0, including 24,244 previously identified genes and 3,054 potentially new genes. Moreover, the analyses revealed that 89.5% of the genes were common in the four lamb groups (Figure 1(a)). In addition, the common DEG number was greater in the compared groups of A-liver^c^-A-liver^w^ and A-liver^c^-H-liver^c^ than the compared groups of A-liver^w^-H-liver^w^ and H-liver^c^-H-liver^w^, which indicated that these two groups had more similar responsive DEGs (Figure 1(b)).

### 3.3. Analysis of the DEGs

After cold exposure, more DEGs were upregulated in the −5°C Altay lambs compared to −5°C Hu lambs, but more DEGs were downregulated in the −5°C Hu lambs compared to 20°C Hu lambs (Figure 1(c)).

**Figure 2:**
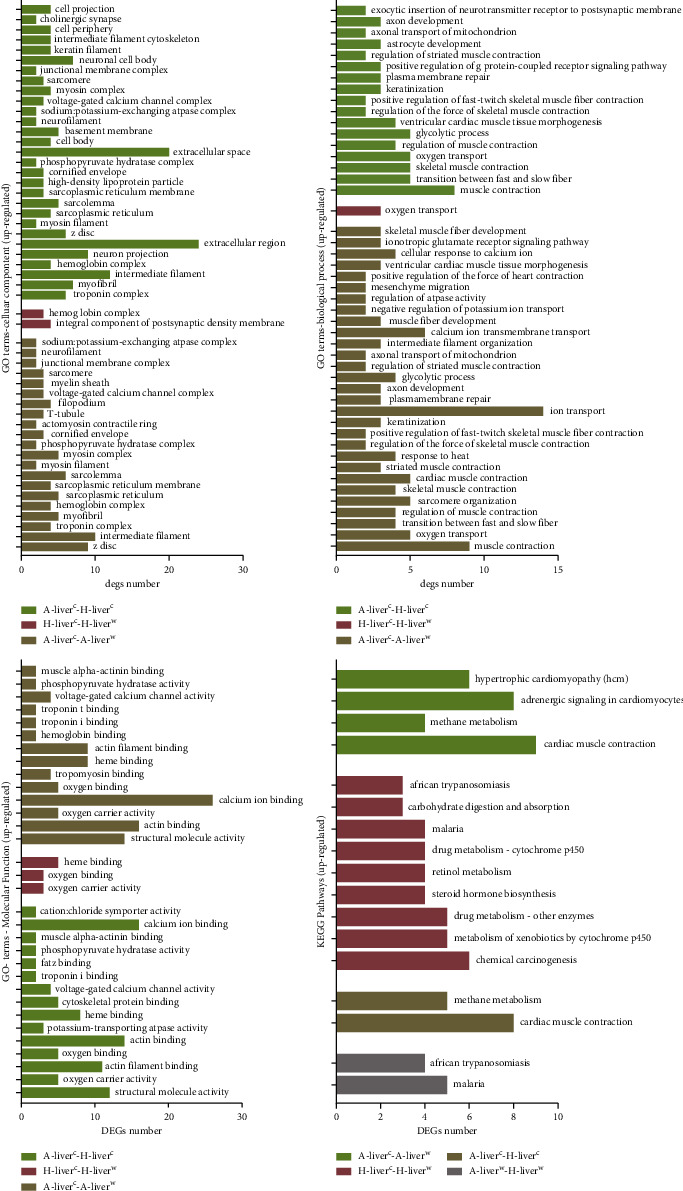
The significant upregulated GO terms and KEGG pathways of DEGs in the liver. A-liver^c^-A-liver^w^: liver of −5°C Altay lambs (*n* = 5) compared to 20°C Altay lambs (*n* = 4); H-liver^c^-H-liver^w^: liver of −5°C Hu lambs (*n* = 6) compared to 20°C Hu lambs (*n* = 4); A-liver^c^-H-liver^c^: liver of −5°C Altay lambs compared to −5°C Hu lambs; A-liver^w^-H-liver^w^: liver of 20°C Altay lambs compared to 20°C Hu lambs. The screen of significant enrichment is based on *q* value < 0.05. The green columns represent the group of A-liver^w^-A-liver^c^, the red columns represent the group of H-liver^w^-H-liver^c^, the khaki columns represent the group of H-liver^c^-A-liver^c^, and the gray columns represent the group of H-liver^w^-A-liver^w^.

According to the classification of GO terms, DEGs were clustered into molecular function (MF), cellular component (CC), and biological process (BP). The selected significant GO terms' annotation is presented in Table S2, and upregulated GO terms are presented in Figure 2. The CC GO category in the cold-exposed Altay lambs was related to muscle contraction, MF GO category was related to the regulation of the binding of muscle-related proteins, and BP GO category was related to muscle contraction, but GO terms in the cold-exposed Hu and Altay lambs differed and were related to the hemoglobin complex in the CC GO category, oxygen carrier activity in the MF GO category, and oxygen transport in the BP GO category. For the downregulated GO terms, hormone activity and lipid binding were enriched in the −5°C Altay lambs compared to 20°C Altay lambs, but chemokine activity and Ca^2+^ binding were enriched in the −5°C Hu lambs compared to 20°C Hu lambs.

Upregulated KEGG pathways in the −5°C Altay lambs compared to 20°C Altay lambs were enriched in cardiac muscle contraction, methane metabolism, and adrenergic signaling in cardiomyocyte pathways. Cardiac muscle contraction and methane metabolism pathways were also upregulated in the −5°C Altay lambs compared to −5°C Hu lambs, but there was no difference in the metabolism pathway in the 20°C Altay lambs compared to 20°C Hu lambs. In contrast to upregulated KEGG pathways, the immune, disease, and metabolism-related pathways were downregulated in the 20°C Altay lambs compared to 20°C Hu lambs. The selected significant KEGG pathways are presented in Table S3.

**Figure 3:**
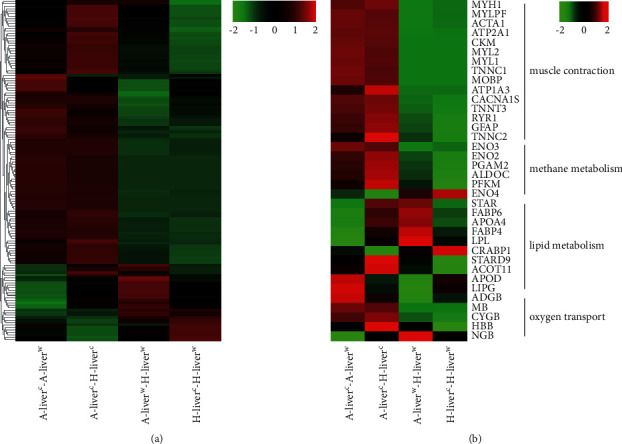
The heatmap of DEGs in the liver. A-liver^c^-A-liver^w^: liver of −5°C Altay lambs (*n* = 5) compared to 20°C Altay lambs (*n* = 4); H-liver^c^-H-liver^w^: liver of −5°C Hu lambs (*n* = 6) compared to 20°C Hu lambs (*n* = 4); A-liver^c^-H-liver^c^: liver of −5°C Altay lambs compared to −5°C Hu lambs; A-liver^w^-H-liver^w^: liver of 20°C Altay lambs compared to 20°C Hu lambs. (a) A heatmap of top 50 DEGs. (b) A heatmap of candidate DEGs. The cluster analysis of gene expression is based on log2 FPKM data. The red color represents higher expression, and the green color represents lower expression.

The heatmap of the top 50 DEGs of the liver exhibited reversed expression trends in the two breeds under cold exposure (Figure 3(a)). The top 50 DEGs of the liver at different temperatures in Altay and Hu lambs are presented in Table S4. The DEGs in the −5°C Hu lambs compared to 20°C Hu lambs and the two breeds at 20°C were primarily downregulated, but DEGs in the −5°C Altay lambs compared to 20°C Altay lambs and compared to −5°C Hu lambs were primarily upregulated. These DEGs were related to energy metabolism, muscle development, and Ca^2+^ binding, whereas the downregulated DEGs in the −5°C Altay lambs compared to 20°C Altay lambs were related to lipid metabolism. We clustered the DEGs, which were enriched in the significant GO and KEGG pathways, including muscle contraction, methane metabolism, lipid metabolism, and oxygen transport (Figure 3(b)). The candidate genes related to muscle contraction, methane metabolism, and oxygen transport were upregulated significantly in the cold-exposed Altay lambs, and almost all of the candidate genes were downregulated in the cold-exposed Hu lambs. Lipid metabolism-related genes displayed large differences between breeds.

### 3.4. Validation of RNA-Seq Results by RT-qPCR

**Figure 4:**
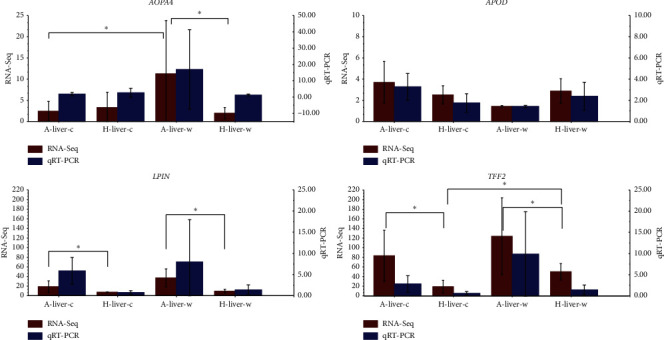
Expression levels of four candidate genes from RT-PCR and RNA-seq. A-liver^w^ and A-liver^c^: liver of Altay lambs under thermoneutral (20°C; *n* = 4) and cold exposure (−5°C; *n* = 5); H-liver^w^ and H-liver^c^: liver of Hu lambs under thermoneutral (20°C; *n* = 4) and cold exposure (−5°C) (*n* = 6). The *X*-axis represents the tissue of the liver; the *Y*-axis on the left represents the relative gene FPKM levels of RNA-seq by columns and bars; the *Y*-axis on the right represents the relative gene expression levels of RT-PCR by columns and bars. ^∗^*P* < 0.05.

Four selected DEGs, namely, *APOD*, *APOA4*, *LPIN*, and *TFF2*, were used to verify RNA-seq by RT-qPCR, and all were in agreement. The four genes were chosen for RT-qPCR because the FPKM of these genes was relatively high, and the genes related to lipid and energy metabolism displayed a high correlation with our study. The relative expression levels of these genes from liver tissue in all four groups are presented in Figure 4.

## 4. Discussion

A number of studies have reported that the liver has a key function in NST. For example, it was reported that the liver provided approximately 44% of the total metabolic energy with cold acclimation in short-tailed opossums [21], oxidation capacity of the liver of ducks increased by 40% after cold adaptation, and with cold adaptation, the liver provided BAT with glucose and fatty acids from very-low-density lipoproteins (VLDLs), which contributed to heat production [22]. In a recent study on the transcriptome profiles of cold-adapted Mongolian sheep [23], cold exposure induced postponing cell senescence in the liver, but no direct evidence of liver involvement in thermogenesis was reported. Therefore, to date, little is known about transcriptome profiles of the liver of lambs when exposed to cold as most studies have been done on rodents and humans. Molecular genetic studies can provide new insights in understanding the mechanisms underlying the tolerance of lambs to cold exposure.

### 4.1. Altay and Hu Sheep Displayed Breed Differences

A large number of downregulated DEGs were enriched in the first level of KEGG pathways, including diseases, organismal systems, and metabolism pathways in Altay lambs compared to Hu lambs. These genetic differences between sheep breeds occurred, especially in immune responses. The DEGs *CD40* and CD40 ligand (*CD40L*) were downregulated in Altay compared to Hu lambs and were enriched in all the top 5 KEGG pathways related to immune processes. *CD40* and *CD40L* regulate inflammatory processes through secondary messengers [24]. By regulating the transcription of different downstream factors, they mediate the immune signal transduction due to *Leishmania* infection [25] and oxidation stress [26]. Enrichment of downregulated genes in immune-related pathways indicated that Altay lambs have stronger immune resistance than Hu lambs. This could be explained by their different backgrounds and adaptations as Altay sheep are indigenous to Xinjiang Altay, have adapted well to the long, cold winters and sparse forage of low protein content, and are resistant to diseases, whereas Hu sheep are typically raised in warm, humid areas and are bred for high reproductive rates.

### 4.2. Cold-Exposed Altay Lambs Enhance Muscle ST and NST through Liver Regulation

The GO analysis indicated that cold-exposed Altay lambs mobilized several terms in the liver related to muscle regulation, such as muscle contraction, transition between fast and slow fibers, and cardiac muscle contraction. These terms were all upregulated. In addition, the cardiac muscle contraction pathway was enriched, and the methane metabolism pathway was upregulated. A large number of DEGs that were focused on muscle contraction, including actin alpha 1 (*ACTA1*), myosin light chain, phosphorylatable, fast skeletal muscle (*MYLPF*), myosin heavy chain 1 (*MYH1*), myosin heavy chain 2 (*MYH2*), myosin light chain 1 (*MYL1*), myosin light chain 2 (*MYL2*), troponin C1, slow skeletal and cardiac type (*TNNC1*), troponin C2, fast skeletal type (*TNNC2*), and troponin T3, fast skeletal type (*TNNT3*), were upregulated in the top 50 DEGs in cold-exposed Altay lambs. These highly expressed genes in the liver of cold-exposed Altay lambs responded to cold stimuli, which indicated that cold exposure enhanced liver-mediated muscle metabolism, including muscle contraction and transition between fast and slow fibers. Studies have reported that the liver has a close metabolic relationship with the muscle and that it could regulate heat production by stimulating muscular activity [27, 28]. In the early stage of cold exposure, ST, through skeletal muscle contraction, plays a major role in thermoregulation, but this is temporary [29]. Recent studies have demonstrated that the skeletal muscle is not only involved in ST but also in NST [30, 31]. In the present study, sarco-endoplasmic reticulum Ca^2+^-ATPase 1 (*ATP2A1*, *SERCA1*) and sarcolipin (*SLN*) were upregulated in the cold-exposed Altay lambs, and the GO terms of the Ca^2+^ transmembrane transport and sarcoplasmic reticulum were activated. The skeletal muscle activates *SLN* by regulating Ca^2+^-ATPase in the endoplasmic reticulum (ER) to produce heat by muscular NST [28, 31, 32]. *ATP2A1* (*SERCA1*), a key enzyme in pumping Ca^2+^, converts part of the energy into heat [33, 34], and *ATP2A1* binds with *SLN*, in the presence of Ca^2+^, and promotes uncoupling of the ATP2A pump [35, 36]. There is evidence that all energy from uncoupled ATP hydrolysis is converted into heat [37], which would suggest that the liver in Altay lambs regulates muscular ST and NST in response to cold exposure. In addition, in the current study, creatine kinase (*CKM*) was the top upregulated DEG in cold-exposed Altay lambs and was enriched in the GO term in response to heat. *CKM* triggers the phosphorylation of creatine from mitochondrial ATP, which liberates local ADP, and then, phosphocreatine (Pcr) is hydrolyzed to creatine to initiate another round of the futile creatine cycle [38]. The Pcr circuit plays an important role in cells that are both excitable and require a high thermodynamic efficiency, such as myocytes. The circuit can transmit chemical energy to the required sites of high ATP demand through cellular distribution of creatine kinase [39, 40].

### 4.3. Cold-Exposed Altay Lambs Enhance Liver Metabolism

Interestingly, the methane metabolism pathway was upregulated in cold-exposed Altay lambs. Related DEGs, including phosphofructokinase, muscle (*PFKM*), aldolase, fructose-bisphosphate C (*ALDOC*), phosphoglycerate mutase 2 (*PGAM2*), enolase 2 (*ENO2*), enolase 3 (*ENO3*), and enolase 4 (*ENO4*), were also enriched and involved in the GO term of glycolytic processes. *PFKM* is the limiting enzyme of the glycolytic process and represents a major control point in the metabolism of glucose [41]. The function of *PFKM* was reported to be associated with the growth of methane-producing bacteria. *ENOs* catalyze the interconversion of 2-phosphoglycerate to phosphoenolpyruvate, which is the second of the two high-energy intermediates that generate ATP in glycolysis [42, 43]. Consequently, cold exposure could result in an increase in glucose metabolism and methane production in Altay lambs. However, cold exposure did not result in changes in rumen methane transcriptome profiles in Altay lambs (unpublished data). Studies have shown that high temperature could reduce methane production in sheep [44], but whether low temperature increases methane emission needs further research.

We found that the GO terms of oxygen transport, oxygen carrier activity, oxygen binding, and KEGG pathway of cardiac muscle contraction were enriched in Altay lambs. Enzyme activity in the citric acid cycle (TCA cycle) was reported to be directly proportional to the rate of oxygen utilization by muscle cells as a function of the increase in ATP requirement for myocardial contraction [45]. Apparently, these 6 candidate genes are related to the glycolytic pathway and TCA cycle to produce energy for thermogenesis. Several DEGs involved in lipid metabolism, such as *APOD* and endothelial lipase (*LIPG*), were upregulated in the cold-exposed Altay lambs compared to 20°C Altay lambs. *LIPG* clears the circulation of high-density lipoproteins and provides lipid precursors for lipid synthesis [46, 47], while the *APOA4* gene is a major constituent of high-density lipoprotein particles [48] and is downregulated in the cold-exposed Altay lambs compared to 20°C Altay lambs. This indicated that cold exposure reduced the circulation of high-density lipoproteins, which resulted in a reduction in transport of cholesterol to the liver. Furthermore, *APOD* not only influences lipid metabolism but also plays an important role in glucose homeostasis [49, 50]. In addition, *TFF2*, as a regulator of energy metabolism [51], was upregulated in the cold-exposed Altay lambs compared to −5°C Hu lambs. These results indicated that cold exposure influenced the cholesterol transport in the liver of Altay lambs and enhanced glycolysis in the liver to generate heat.

### 4.4. The Liver May Not Be the Main Thermogenic Tissue for Hu Lambs

Through the cluster of candidate DEGs, almost all candidate genes were downregulated in the cold-exposed Hu lambs, which means that the liver may not be the main thermogenic tissue. Compared to cold-exposed Altay lambs, the response of the liver in Hu lambs to cold exposure was focused mainly on immune diseases and immune system pathways. The gene transcriptional expression level had smaller differences between breeds of 20°C lamb group. In a previous study (unpublished data), cold-exposed Hu lambs relied mainly on tail fat for heat production, rather than typical thermogenic tissues such as the liver and muscles. In the present study, cellular retinoic acid-binding protein type 1 (*CRABP1*) was upregulated in the −5°C Hu lambs compared to 20°C Hu lambs, but downregulated in the −5°C Altay lambs compared to −5°C Hu lambs, whereas acyl-coA thioesterase 11 (*ACOT11*) regulation displayed an opposite trend. *CRABP1* is one of the carrier proteins of retinoic acid and was reported to be associated with fat accumulation in adipose tissue in mice [52]. *ACOT11* was reported to limit the oxidation of lipid droplet-derived fatty acids, possibly by regulating the availability of substrates to be used for *β*-oxidation and uncoupling [53–55]. These results indicate that cold exposure mobilizes adipose tissue as the main heat-producing tissue in Hu lambs and could explain why cold exposure did not cause enrichment and upregulation of energy metabolism-related pathways and genes in their liver.

## 5. Conclusions

Transcriptome sequencing of the liver of cold-exposed Altay and Hu lambs revealed different thermogenic pathways between breeds. Cold exposure induced thermogenesis in Altay lambs and activated the liver-regulated muscle contraction pathway related to ST of muscles. Eight candidate genes, namely, *ACTA1*, *MYH1*, *MYH2*, *MYL1*, *MYL2*, *TNNC1*, *TNNC2*, and *TNNT3*, were upregulated in response to cold. The Ca^2+^ signal pathway and creatine cycle were also activated, and 3 candidate genes, including *ATP2A1*, *SLN*, and *CKM*, were involved and upregulated in muscular NST. In addition, 6 candidate genes related to methane metabolism, *PFKM*, *ALDOC*, *PGAM2*, *ENO2*, *ENO3*, and *ENO4*, were upregulated in the liver of cold-exposed Altay lambs. However, in the cold-exposed Hu lambs, it appears that the liver is not the main tissue for thermogenesis, but has a much lesser role when compared to Altay lambs. In summary, Altay and Hu lambs have different regulation mechanisms in response to cold exposure, which are likely manifested in breed differences in cold resistance.

## Figures and Tables

**Table 1:** Primer sequences of genes for RT-PCR validation.

Gene name	Primer sequences (5′–3′)	Products' size (bp)	Tm (^o^C)
*APOD*	GTGAACTGTCCCGAGTCCAT	121	60
	GCTCTGGTGGTTTGGTTTGT		
*APOA4*	TGTCTGTCTGTCCCAAAGCA	81	60
	TGAGAAGCGAGAGGTAGCATC		
*TFF2*	AAGTGCTGCTTCTCCGACA	158	60
	TCCGAAACTGTAAGATGGTGAG		
*LPIN*	GATGAAAGAGTCCAGCCCAT	200	60
	CCAAAGCCTCAATGTCGTCT		
*β-Actin*	AGCCTTCCTTCCTGGGCATGGA	113	60
	GGACAGCACCGTGTTGGCGTAGA		

## Data Availability

The data are available confidentially to editors and reviewers, and all transcriptome data were submitted to the NCBI Sequence Read Archive (SRA series accession: PRJNA639638).

## Supplementary Materials

Supplementary Materials Supplementary Material 1: Figure S1: CPCoA analyses of all samples and sequencing quality in the liver of Altay and Hu lambs. Supplementary Material 2: Table S1: summary of RNA-seq results. Supplementary Material 3: Table S2: GO terms significantly enriched in the liver at different temperatures in Altay and Hu lambs. Supplementary Material 4: Table S3: KEGG pathways significantly enriched in the liver at different temperatures in Altay and Hu lambs. Supplementary Material 5: Table S4: top 50 DEGs in the liver at different temperatures in Altay and Hu lambs. *s*
