# Supplementary material for: Transcriptome Profiles of the Liver in Two Cold-Exposed Sheep Breeds Revealed Different Mechanisms and Candidate Genes for Thermogenesis
Source: Genet Res (Camb). 2021 Aug 10;2021:5510297. doi: 10.1155/2021/5510297 (PMC9364924; doi:10.1155/2021/5510297)
Supplement: Supplementary Materials — Supplementary Material 1: Figure S1: CPCoA analyses of all samples and sequencing quality in the liver of Altay and Hu lambs. Supplementary Material 2: Table S1: summary of RNA-seq results. Supplementary Material 3: Table S2: GO terms significantly enriched in the liver at different temperatures in Altay and Hu lambs. Supplementary Material 4: Table S3: KEGG pathways significantly enriched in the liver at different temperatures in Altay and Hu lambs. Supplementary Material 5: Table S4: top 50 DEGs in the liver at different temperatures in Altay and Hu lambs. s [file 5510297.f1.zip › 5510297.f1/Figure S1 (1).pdf]

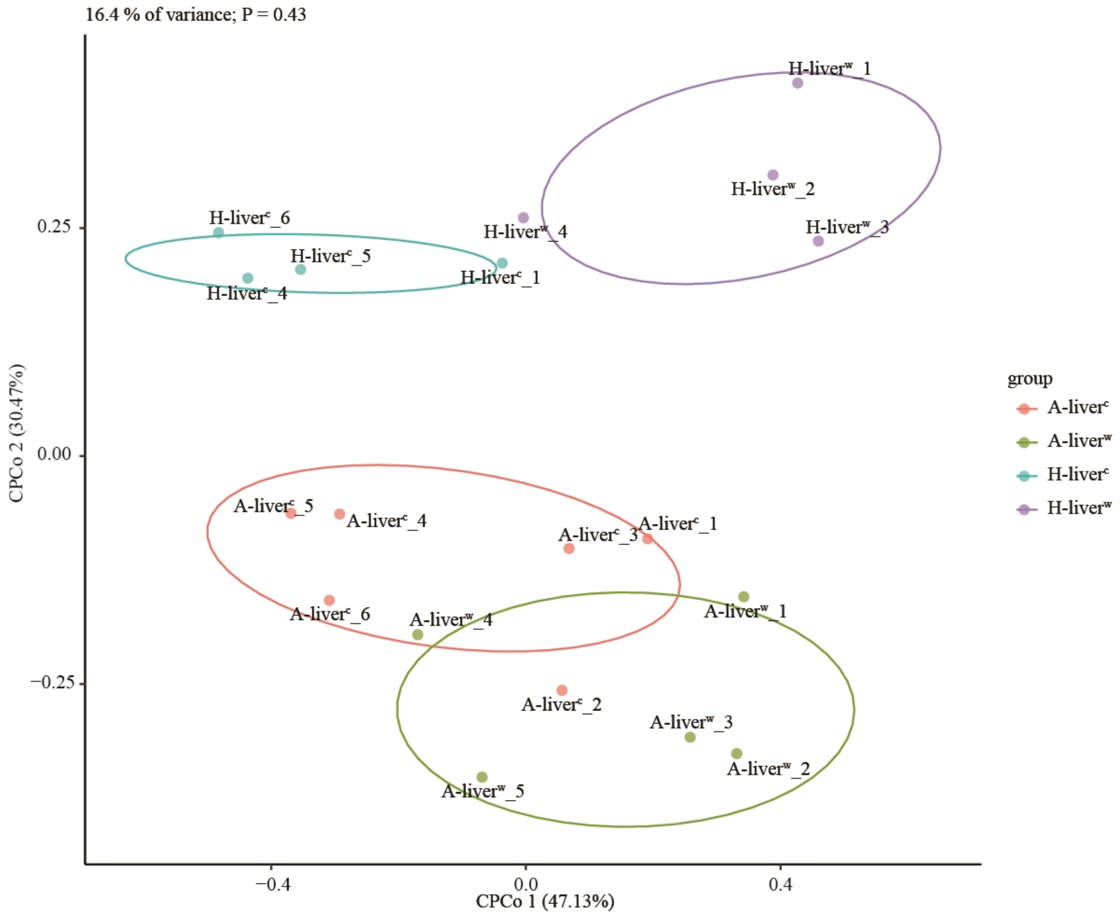

Figure S1. CPCoA analyses of all samples and sequencing quality in the liver of Altay and Hu lambs. A-liver^c and H-liver^c: liver of cold-exposed (-5 °C) Altay and Hu lambs; A-liver^w and H-liver^w: liver of warm-exposed (20 °C) Altay and Hu lambs; red points represent liver of cold-exposed (-5 °C) Altay lambs, green points represent liver of warm-exposed (20 °C) Hu lambs, blue points represent liver of warm-exposed (-5 °C) Altay lambs and purple points represent liver of warm-exposed (20 °C) Hu lambs.
