# Supplementary material for: Transcriptome Profiles of the Liver in Two Cold-Exposed Sheep Breeds Revealed Different Mechanisms and Candidate Genes for Thermogenesis
Source: Genet Res (Camb). 2021 Aug 10;2021:5510297. doi: 10.1155/2021/5510297 (PMC9364924; doi:10.1155/2021/5510297)
Supplement: Supplementary Materials — Supplementary Material 1: Figure S1: CPCoA analyses of all samples and sequencing quality in the liver of Altay and Hu lambs. Supplementary Material 2: Table S1: summary of RNA-seq results. Supplementary Material 3: Table S2: GO terms significantly enriched in the liver at different temperatures in Altay and Hu lambs. Supplementary Material 4: Table S3: KEGG pathways significantly enriched in the liver at different temperatures in Altay and Hu lambs. Supplementary Material 5: Table S4: top 50 DEGs in the liver at different temperatures in Altay and Hu lambs. s [file 5510297.f1.zip › 5510297.f1/Table S1.docx]

Table S1. Summary of RNA-seq results.

| Reads |  | A-liver^c^ | H-liver^c^ | A-liver^w^ | H-liver^w^ |
| --- | --- | --- | --- | --- | --- |
| Total | Raw reads | 45,308,108 | 43,622,042 | 46,476,543 | 47,349,509 |
|  | Clean reads | 43,552,826 | 42,647,691 | 44,172,097 | 44,787,574 |
|  | N | 1,750,794 | 967,517 | 2,299,714 | 2,561,330 |
|  | Adapter | 0 | 0 | 0 | 0 |
|  | Low quality | 4,570 | 6,939 | 4,118 | 2,328 |
| Matches to genome | Total mapped reads | 41,512,258 | 40,737,341 | 42,011,198 | 42,612,500 |
|  | Unique match | 37,053,023 | 36,218,569 | 37,659,047 | 38,719,462 |
| Matches to genes | Total mapped reads | 31,460,438 | 30,370,831 | 31,923,461 | 32,971,805 |
|  | Unique match | 28,429,299 | 27,410,935 | 28,915,893 | 29,813,300 |

A-liver^c^ and H-liver^c^: liver of cold-exposed (-5 ℃) Altay and Hu lambs; A-liver^w^ and H-liver^w^: liver of thermo-neutral-exposed (20 ℃) Altay and Hu lambs;
