# Supplementary material for: Transcriptome Profiles of the Liver in Two Cold-Exposed Sheep Breeds Revealed Different Mechanisms and Candidate Genes for Thermogenesis
Source: Genet Res (Camb). 2021 Aug 10;2021:5510297. doi: 10.1155/2021/5510297 (PMC9364924; doi:10.1155/2021/5510297)
Supplement: Supplementary Materials — Supplementary Material 1: Figure S1: CPCoA analyses of all samples and sequencing quality in the liver of Altay and Hu lambs. Supplementary Material 2: Table S1: summary of RNA-seq results. Supplementary Material 3: Table S2: GO terms significantly enriched in the liver at different temperatures in Altay and Hu lambs. Supplementary Material 4: Table S3: KEGG pathways significantly enriched in the liver at different temperatures in Altay and Hu lambs. Supplementary Material 5: Table S4: top 50 DEGs in the liver at different temperatures in Altay and Hu lambs. s [file 5510297.f1.zip › 5510297.f1/Table S2.docx]

| Table S2. GO terms significantly enriched in liver at different temperatures in group of A-liver^c^-A-liver^w^. | | | | |
| --- | --- | --- | --- | --- |
| GO_C Term ID | GO_C Term Desc | GO_C Term Level1 | Term Candidate Gene Num | Q value |
| GO:0005576 | extracellular region | cellular_component | 33 | 5.01E-04 |
| GO:0005882 | intermediate filament | cellular_component | 11 | 5.01E-04 |
| GO:0016529 | sarcoplasmic reticulum | cellular_component | 6 | 5.01E-04 |
| GO:0030018 | Z disc | cellular_component | 9 | 5.01E-04 |
| GO:0005861 | troponin complex | cellular_component | 4 | 8.98E-04 |
| GO:0005833 | hemoglobin complex | cellular_component | 4 | 0.00177365 |
| GO:0005891 | voltage-gated calcium channel complex | cellular_component | 5 | 0.00177365 |
| GO:0030016 | myofibril | cellular_component | 5 | 0.00177365 |
| GO:0033017 | sarcoplasmic reticulum membrane | cellular_component | 4 | 0.004643405 |
| GO:0016459 | myosin complex | cellular_component | 6 | 0.007230426 |
| GO:0042383 | sarcolemma | cellular_component | 6 | 0.008637219 |
| GO:0032982 | myosin filament | cellular_component | 2 | 0.010645446 |
| GO:0030175 | filopodium | cellular_component | 5 | 0.019583453 |
| GO:0000015 | phosphopyruvate hydratase complex | cellular_component | 2 | 0.046429255 |
| GO:0005826 | actomyosin contractile ring | cellular_component | 2 | 0.046429255 |
| GO:0034364 | high-density lipoprotein particle | cellular_component | 3 | 0.046429255 |
| GO_F Term ID | GO_F Term Desc | GO_F Term Level1 | Term Candidate Gene Num | Q value |
| GO:0005344 | oxygen carrier activity | molecular_function | 6 | 6.27E-05 |
| GO:0019825 | oxygen binding | molecular_function | 6 | 8.81E-05 |
| GO:0005198 | structural molecule activity | molecular_function | 14 | 3.77E-04 |
| GO:0051015 | actin filament binding | molecular_function | 12 | 8.88E-04 |
| GO:0005245 | voltage-gated calcium channel activity | molecular_function | 6 | 0.002082558 |
| GO:0020037 | heme binding | molecular_function | 10 | 0.004431307 |
| GO:0003779 | actin binding | molecular_function | 15 | 0.007260401 |
| GO:0008556 | potassium-transporting ATPase activity | molecular_function | 3 | 0.009635904 |
| GO:0004465 | lipoprotein lipase activity | molecular_function | 2 | 0.01848777 |
| GO:0052689 | carboxylic ester hydrolase activity | molecular_function | 4 | 0.01848777 |
| GO:0005262 | calcium channel activity | molecular_function | 6 | 0.02131381 |
| GO:0005216 | ion channel activity | molecular_function | 11 | 0.03415834 |
| GO:0005244 | voltage-gated ion channel activity | molecular_function | 7 | 0.03415834 |
| GO:0005509 | calcium ion binding | molecular_function | 23 | 0.03415834 |
| GO:0031013 | troponin I binding | molecular_function | 2 | 0.03415834 |
| GO:0051373 | FATZ binding | molecular_function | 2 | 0.03415834 |
| GO:0008092 | cytoskeletal protein binding | molecular_function | 5 | 0.03952008 |
| GO:0005179 | hormone activity | molecular_function | 7 | 0.04014608 |
| GO_P Term ID | GO_P Term Desc | GO_P Term Level1 | Term Candidate Gene Num | Q value |
| GO:0006936 | muscle contraction | biological_process | 9 | 3.68E-07 |
| GO:0015671 | oxygen transport | biological_process | 6 | 5.93E-05 |
| GO:0006811 | ion transport | biological_process | 24 | 2.22E-04 |
| GO:0014883 | transition between fast and slow fiber | biological_process | 4 | 0.002361916 |
| GO:0006941 | striated muscle contraction | biological_process | 4 | 0.003711129 |
| GO:0006937 | regulation of muscle contraction | biological_process | 4 | 0.005466693 |
| GO:0045214 | sarcomere organization | biological_process | 5 | 0.01381084 |
| GO:0003009 | skeletal muscle contraction | biological_process | 4 | 0.01525586 |
| GO:0070588 | calcium ion transmembrane transport | biological_process | 8 | 0.0428129 |
| GO:0014728 | regulation of the force of skeletal muscle contraction | biological_process | 2 | 0.04401714 |
| GO:0030644 | cellular chloride ion homeostasis | biological_process | 2 | 0.04401714 |
| GO:0031448 | positive regulation of fast-twitch skeletal muscle fiber contraction | biological_process | 2 | 0.04401714 |
| GO:0060048 | cardiac muscle contraction | biological_process | 5 | 0.04401714 |

| Table S2. GO terms significantly enriched in liver at different temperatures in group of H-liver^c^-H-liver^w^. | | | | |
| --- | --- | --- | --- | --- |
| GO_C Term ID | GO_C Term Desc | GO_C Term Level1 | Term Candidate Gene Num | Q value |
| GO:0005576 | extracellular region | cellular_component | 58 | 8.89E-06 |
| GO:0016020 | membrane | cellular_component | 256 | 1.70E-04 |
| GO:0016021 | integral component of membrane | cellular_component | 236 | 3.44E-04 |
| GO:0005615 | extracellular space | cellular_component | 49 | 6.04E-04 |
| GO:0009897 | external side of plasma membrane | cellular_component | 19 | 0.001811464 |
| GO:0042612 | MHC class I protein complex | cellular_component | 5 | 0.001811464 |
| GO:0031012 | extracellular matrix | cellular_component | 14 | 0.01771084 |
| GO:0042613 | MHC class II protein complex | cellular_component | 5 | 0.02581305 |
| GO:0008091 | spectrin | cellular_component | 3 | 0.02829397 |
| GO:0009986 | cell surface | cellular_component | 26 | 0.03717853 |
| GO_F Term ID | GO_F Term Desc | GO_F Term Level1 | Term Candidate Gene Num | Q value |
| GO:0008009 | chemokine activity | molecular_function | 9 | 0.007610543 |
| GO:0008237 | metallopeptidase activity | molecular_function | 16 | 0.008558032 |
| GO:0005509 | calcium ion binding | molecular_function | 42 | 0.026182856 |
| GO_P Term ID | GO_P Term Desc | GO_P Term Level1 | Term Candidate Gene Num | Q value |
| GO:0006955 | immune response | biological_process | 29 | 3.65E-06 |
| GO:0060326 | cell chemotaxis | biological_process | 11 | 0.01430871 |
| GO:0002474 | antigen processing and presentation of peptide antigen via MHC class I | biological_process | 5 | 0.0238429 |
| GO:0042113 | B cell activation | biological_process | 6 | 0.0238429 |
| GO:0045064 | T-helper 2 cell differentiation | biological_process | 3 | 0.0385466 |

| Table S2. GO terms significantly enriched in liver at different temperatures in group of A-liver^c^-H-liver^c^. | | | | |
| --- | --- | --- | --- | --- |
| GO_C Term ID | GO_C Term Desc | GO_C Term Level1 | Term Candidate Gene Num | Q value |
| GO:0005861 | troponin complex | cellular_component | 6 | 1.05E-06 |
| GO:0030016 | myofibril | cellular_component | 7 | 1.15E-05 |
| GO:0005882 | intermediate filament | cellular_component | 12 | 7.98E-05 |
| GO:0005604 | basement membrane | cellular_component | 9 | 9.31E-04 |
| GO:0005576 | extracellular region | cellular_component | 32 | 0.001158529 |
| GO:0000015 | phosphopyruvate hydratase complex | cellular_component | 3 | 0.001679766 |
| GO:0005615 | extracellular space | cellular_component | 30 | 0.001679766 |
| GO:0005833 | hemoglobin complex | cellular_component | 4 | 0.001679766 |
| GO:0043005 | neuron projection | cellular_component | 10 | 0.00353858 |
| GO:0042383 | sarcolemma | cellular_component | 6 | 0.009977898 |
| GO:0032982 | myosin filament | cellular_component | 2 | 0.01179776 |
| GO:0016020 | membrane | cellular_component | 136 | 0.01454773 |
| GO:0005891 | voltage-gated calcium channel complex | cellular_component | 4 | 0.01611793 |
| GO:0016529 | sarcoplasmic reticulum | cellular_component | 4 | 0.02492814 |
| GO:0030018 | Z disc | cellular_component | 6 | 0.02492814 |
| GO:0016021 | integral component of membrane | cellular_component | 123 | 0.04067486 |
| GO:0033017 | sarcoplasmic reticulum membrane | cellular_component | 3 | 0.04067486 |
| GO:0034364 | high-density lipoprotein particle | cellular_component | 3 | 0.04067486 |
| GO:0001533 | cornified envelope | cellular_component | 3 | 0.04819044 |
| GO_F Term ID | GO_F Term Desc | GO_F Term Level1 | Term Candidate Gene Num | Q value |
| GO:0005509 | calcium ion binding | molecular_function | 35 | 1.97E-05 |
| GO:0005198 | structural molecule activity | molecular_function | 15 | 4.19E-04 |
| GO:0005344 | oxygen carrier activity | molecular_function | 5 | 0.00129942 |
| GO:0019825 | oxygen binding | molecular_function | 5 | 0.002183986 |
| GO:0004634 | phosphopyruvate hydratase activity | molecular_function | 3 | 0.004770178 |
| GO:0005523 | tropomyosin binding | molecular_function | 4 | 0.007069464 |
| GO:0003779 | actin binding | molecular_function | 16 | 0.00758025 |
| GO:0005245 | voltage-gated calcium channel activity | molecular_function | 5 | 0.02561705 |
| GO:0051015 | actin filament binding | molecular_function | 10 | 0.02561705 |
| GO:0020037 | heme binding | molecular_function | 9 | 0.03174894 |
| GO_P Term ID | GO_P Term Desc | GO_P Term Level1 | Term Candidate Gene Num | Q value |
| GO:0006936 | muscle contraction | biological_process | 8 | 1.71E-05 |
| GO:0014883 | transition between fast and slow fiber | biological_process | 5 | 8.42E-05 |
| GO:0003009 | skeletal muscle contraction | biological_process | 5 | 0.001437671 |
| GO:0015671 | oxygen transport | biological_process | 5 | 0.001437671 |
| GO:0006096 | glycolytic process | biological_process | 6 | 0.007938633 |
| GO:0006937 | regulation of muscle contraction | biological_process | 4 | 0.007938633 |
| GO:0048747 | muscle fiber development | biological_process | 4 | 0.04142182 |

| Table S2. GO terms significantly enriched in liver at different temperatures in group of A-liver^w^-H-liver^w^. | | | | |
| --- | --- | --- | --- | --- |
| GO_C Term ID | GO_C Term Desc | GO_C Term Level1 | Term Candidate Gene Num | Q value |
| GO:0005576 | extracellular region | cellular_component | 64 | 2.45E-08 |
| GO:0016021 | integral component of membrane | cellular_component | 248 | 1.35E-06 |
| GO:0016020 | membrane | cellular_component | 261 | 8.13E-06 |
| GO:0009897 | external side of plasma membrane | cellular_component | 23 | 1.16E-05 |
| GO:0031012 | extracellular matrix | cellular_component | 18 | 1.06E-04 |
| GO:0005615 | extracellular space | cellular_component | 48 | 6.84E-04 |
| GO:0009986 | cell surface | cellular_component | 31 | 6.84E-04 |
| GO:0031430 | M band | cellular_component | 4 | 0.01571505 |
| GO:0005581 | collagen trimer | cellular_component | 8 | 0.01740888 |
| GO:0005833 | hemoglobin complex | cellular_component | 4 | 0.01875356 |
| GO:0042613 | MHC class II protein complex | cellular_component | 5 | 0.01875356 |
| GO:0030017 | sarcomere | cellular_component | 5 | 0.03862646 |
| GO:0005604 | basement membrane | cellular_component | 9 | 0.03989353 |
| GO:0005584 | collagen type I trimer | cellular_component | 2 | 0.0407966 |
| GO:0008076 | voltage-gated potassium channel complex | cellular_component | 8 | 0.0407966 |
| GO:0032982 | myosin filament | cellular_component | 2 | 0.0407966 |
| GO_F Term ID | GO_F Term Desc | GO_F Term Level1 | Term Candidate Gene Num | Q value |
| GO:0005509 | calcium ion binding | molecular_function | 43 | 0.01255427 |
| GO:0005244 | voltage-gated ion channel activity | molecular_function | 12 | 0.01590808 |
| GO:0008009 | chemokine activity | molecular_function | 8 | 0.01590808 |
| GO:0005216 | ion channel activity | molecular_function | 18 | 0.02160387 |
| GO:0005249 | voltage-gated potassium channel activity | molecular_function | 9 | 0.02160387 |
| GO:0019825 | oxygen binding | molecular_function | 5 | 0.02160387 |
| GO:0045545 | syndecan binding | molecular_function | 3 | 0.02160387 |
| GO:0048248 | CXCR3 chemokine receptor binding | molecular_function | 3 | 0.02160387 |
| GO:0008201 | heparin binding | molecular_function | 11 | 0.02880236 |
| GO:0008237 | metallopeptidase activity | molecular_function | 13 | 0.04656931 |
| GO_P Term ID | GO_P Term Desc | GO_P Term Level1 | Term Candidate Gene Num | Q value |
| GO:0006955 | immune response | biological_process | 27 | 3.95E-05 |
| GO:0007155 | cell adhesion | biological_process | 26 | 0.002743909 |
| GO:0051310 | metaphase plate congression | biological_process | 5 | 0.02431376 |
| GO:0006813 | potassium ion transport | biological_process | 14 | 0.04148054 |
| GO:0034765 | regulation of ion transmembrane transport | biological_process | 12 | 0.04287953 |
