# Supplementary material for: Transcriptome Profiles of the Liver in Two Cold-Exposed Sheep Breeds Revealed Different Mechanisms and Candidate Genes for Thermogenesis
Source: Genet Res (Camb). 2021 Aug 10;2021:5510297. doi: 10.1155/2021/5510297 (PMC9364924; doi:10.1155/2021/5510297)
Supplement: Supplementary Materials — Supplementary Material 1: Figure S1: CPCoA analyses of all samples and sequencing quality in the liver of Altay and Hu lambs. Supplementary Material 2: Table S1: summary of RNA-seq results. Supplementary Material 3: Table S2: GO terms significantly enriched in the liver at different temperatures in Altay and Hu lambs. Supplementary Material 4: Table S3: KEGG pathways significantly enriched in the liver at different temperatures in Altay and Hu lambs. Supplementary Material 5: Table S4: top 50 DEGs in the liver at different temperatures in Altay and Hu lambs. s [file 5510297.f1.zip › 5510297.f1/Table S3.docx]

| Table S3. KEGG pathways significantly enriched in liver at different temperatures in group of A-liver^c^-A-liver^w^. | | | | |
| --- | --- | --- | --- | --- |
| KEGG Pathway Term Desc | KEGG Pathway Term Level1 | KEGG Pathway Term Level2 | Term Candidate Gene Num | Q value |
| Cardiac muscle contraction | Organismal Systems | Circulatory system | 10 | 6.42E-04 |
| Hypertrophic cardiomyopathy (HCM) | Human Diseases | Cardiovascular diseases | 9 | 0.01019586 |

| Table S3. KEGG pathways significantly enriched in liver at different temperatures in group of H-liver^c^-H-liver^w^. | | | | |
| --- | --- | --- | --- | --- |
| KEGG Pathway Term Desc | KEGG Pathway Term Level1 | KEGG Pathway Term Level2 | Term Candidate Gene Num | Q value |
| Cytokine-cytokine receptor interaction | Environmental Information Processing | Signaling molecules and interaction | 34 | 5.33E-06 |
| Cell adhesion molecules (CAMs) | Environmental Information Processing | Signaling molecules and interaction | 23 | 5.33429E-06 |
| Autoimmune thyroid disease | Human Diseases | Immune diseases | 13 | 1.56322E-05 |
| Allograft rejection | Human Diseases | Immune diseases | 11 | 8.67234E-05 |
| Type I diabetes mellitus | Human Diseases | Endocrine and metabolic diseases | 11 | 0.000182148 |
| Graft-versus-host disease | Human Diseases | Immune diseases | 10 | 0.000182148 |
| Primary immunodeficiency | Human Diseases | Immune diseases | 9 | 0.000282304 |
| Intestinal immune network for IgA production | Organismal Systems | Immune system | 9 | 0.003974916 |
| NF-kappa B signaling pathway | Environmental Information Processing | Signal transduction | 12 | 0.01132729 |
| Human T-cell leukemia virus 1 infection | Human Diseases | Infectious diseases: Viral | 21 | 0.01141044 |
| Antigen processing and presentation | Organismal Systems | Immune system | 10 | 0.01494891 |
| Chemokine signaling pathway | Organismal Systems | Immune system | 17 | 0.01951471 |
| Hematopoietic cell lineage | Organismal Systems | Immune system | 12 | 0.01951471 |
| Viral myocarditis | Human Diseases | Cardiovascular diseases | 9 | 0.01951471 |
| Th17 cell differentiation | Organismal Systems | Immune system | 12 | 0.02294038 |
| T cell receptor signaling pathway | Organismal Systems | Immune system | 11 | 0.04247681 |

| Table S3. KEGG pathways significantly enriched in liver at different temperatures in group of A-liver^c^-H-liver^c^. | | | | |
| --- | --- | --- | --- | --- |
| KEGG Pathway Term Desc | KEGG Pathway Term Level1 | KEGG Pathway Term Level2 | Term Candidate Gene Num | Q value |
| Cardiac muscle contraction | Organismal Systems | Circulatory system | 10 | 3.75E-04 |
| Methane metabolism | Metabolism | Energy metabolism | 6 | 0.001675182 |
| Biosynthesis of amino acids | Metabolism | Global and overview maps | 8 | 0.011074993 |
| Carbon metabolism | Metabolism | Global and overview maps | 9 | 0.041584068 |
| Malaria | Human Diseases | Infectious diseases: Parasitic | 6 | 0.041584068 |
| African trypanosomiasis | Human Diseases | Infectious diseases: Parasitic | 5 | 0.046649605 |

| Table S3. KEGG pathways significantly enriched in liver at different temperatures in group of A-liver^w^-H-liver^w^. | | | | |
| --- | --- | --- | --- | --- |
| KEGG Pathway Term Desc | KEGG Pathway Term Level1 | KEGG Pathway Term Level2 | Term Candidate Gene Num | Q value |
| Cytokine-cytokine receptor interaction | Environmental Information Processing | Signaling molecules and interaction | 32 | 1.04E-04 |
| Primary immunodeficiency | Human Diseases | Immune diseases | 10 | 0.000121119 |
| Hematopoietic cell lineage | Organismal Systems | Immune system | 14 | 0.004221705 |
| Autoimmune thyroid disease | Human Diseases | Immune diseases | 10 | 0.004221705 |
| Allograft rejection | Human Diseases | Immune diseases | 9 | 0.004221705 |
| Intestinal immune network for IgA production | Organismal Systems | Immune system | 9 | 0.006043189 |
| Cell adhesion molecules (CAMs) | Environmental Information Processing | Signaling molecules and interaction | 17 | 0.006586253 |
| Type I diabetes mellitus | Human Diseases | Endocrine and metabolic diseases | 8 | 0.032919397 |
| Graft-versus-host disease | Human Diseases | Immune diseases | 7 | 0.039830538 |
| Malaria | Human Diseases | Infectious diseases: Parasitic | 8 | 0.044665696 |
