# Supplementary material for: Transcriptome Profiles of the Liver in Two Cold-Exposed Sheep Breeds Revealed Different Mechanisms and Candidate Genes for Thermogenesis
Source: Genet Res (Camb). 2021 Aug 10;2021:5510297. doi: 10.1155/2021/5510297 (PMC9364924; doi:10.1155/2021/5510297)
Supplement: Supplementary Materials — Supplementary Material 1: Figure S1: CPCoA analyses of all samples and sequencing quality in the liver of Altay and Hu lambs. Supplementary Material 2: Table S1: summary of RNA-seq results. Supplementary Material 3: Table S2: GO terms significantly enriched in the liver at different temperatures in Altay and Hu lambs. Supplementary Material 4: Table S3: KEGG pathways significantly enriched in the liver at different temperatures in Altay and Hu lambs. Supplementary Material 5: Table S4: top 50 DEGs in the liver at different temperatures in Altay and Hu lambs. s [file 5510297.f1.zip › 5510297.f1/Table S4.docx]

| Table S4. Top 50 DEGs in liver at different temperatures in Altay and Hu lambs. | | | | | | | |
| --- | --- | --- | --- | --- | --- | --- | --- |
| A-liver^c^-A-liver^w^ | | | | H-liver^c^-H-liver^w^ | | | |
| Gene ID | Gene Symbol | log2FC | Qvalue | Gene ID | Gene Symbol | log2FC | Qvalue |
| 101112841 | CKM | 10.92799604 | 2.62E-150 | 114113605 | LOC114113605 | 8.865004346 | 3.16E-43 |
| 101107662 | ACTA1 | 10.39469565 | 7.39E-226 | 114117594 | LOC114117594 | -8.592249227 | 3.59E-40 |
| 101118481 | LOC101118481 | 9.941110856 | 0 | 114116451 | LOC114116451 | 8.208881383 | 7.97E-30 |
| 114115573 | LOC114115573 | 8.482794674 | 0 | 114108993 | LOC114108993 | -7.976523285 | 2.93E-28 |
| 100270715 | MYLPF | 8.444571562 | 3.91E-76 | 114112309 | LOC114112309 | -7.824780745 | 7.76E-90 |
| 114112267 | LOC114112267 | -8.406186214 | 7.80E-112 | 114115271 | LOC114115271 | 7.118106294 | 7.51E-16 |
| 442994 | MYH1 | 8.404629745 | 2.12E-74 | 101111100 | CXCL13 | -6.656229853 | 4.27E-49 |
| 101105132 | LOC101105132 | 8.220205173 | 1.06E-33 | 101110285 | CCDC190 | 6.025447802 | 2.66E-08 |
| 101109898 | EEF1A2 | 8.192699262 | 3.59E-33 | 101105132 | LOC101105132 | -5.891327382 | 3.11E-08 |
| 780509 | MB | 8.14608832 | 5.55E-64 | 101122145 | MROH7 | -5.786328253 | 8.09E-163 |
| 101111827 | LOC101111827 | -7.956174483 | 1.51E-24 | 100048994 | CYP11A1 | -5.662802507 | 2.51E-13 |
| 101101869 | MYL1 | 7.791494036 | 3.00E-26 | 101120527 | STRA6 | -5.405004749 | 3.77E-06 |
| 114115623 | LOC114115623 | -7.405830179 | 8.77E-18 | 101119401 | COL22A1 | -5.340874412 | 6.39E-06 |
| 114115271 | LOC114115271 | -7.253603499 | 3.01E-16 | 101120468 | CCL27 | -5.26963233 | 1.12E-05 |
| 114110327 | LOC114110327 | 6.77164927 | 0 | 101112304 | KRT6A | -5.234060092 | 1.47E-05 |
| 101105314 | LOC101105314 | 6.720575772 | 2.37E-80 | 114112166 | LOC114112166 | 5.231257339 | 3.42E-05 |
| 101119079 | LOC101119079 | 6.61605203 | 5.53E-13 | 101113487 | LOC101113487 | -5.203370888 | 1.86E-05 |
| 101113800 | MOBP | 6.590961049 | 8.71E-13 | 100462743 | DUOX2 | -5.129370307 | 3.18E-05 |
| 114117593 | LOC114117593 | 6.549624668 | 1.82E-12 | 101114742 | ST8SIA3 | -5.101713964 | 2.56E-92 |
| 101121072 | PLP1 | 6.479170223 | 1.37E-45 | 114110170 | LRRN4CL | -5.081507655 | 4.46E-05 |
| 101104853 | SLN | 6.312659886 | 8.97E-11 | 101123417 | LOC101123417 | -5.051367795 | 6.01E-09 |
| 105603393 | LOC105603393 | -6.296777443 | 3.81E-09 | 101108767 | CACNA1S | -5.051367795 | 5.48E-05 |
| 101105997 | LOC101105997 | 6.184335789 | 5.92E-10 | 443245 | PGF | -5.051367795 | 5.48E-05 |
| 100196904 | MYL2 | 6.005998548 | 6.48E-09 | 101123372 | LOC101123372 | -4.97275796 | 1.68E-83 |
| 443220 | GFAP | 5.845533876 | 3.96E-74 | 100431098 | SCGB1A1 | -4.968905635 | 9.46E-05 |
| 101108767 | CACNA1S | 5.802465154 | 6.18E-15 | 114108777 | LOC114108777 | 4.926860656 | 1.05E-120 |
| 114115658 | LOC114115658 | 5.790846071 | 0.003283042 | 101122151 | ITLN2 | -4.923211443 | 1.27E-04 |
| 114110308 | LOC114110308 | 5.531565682 | 0 | 114110288 | LOC114110288 | -4.822549104 | 2.34E-04 |
| 100127216 | TNNC2 | 5.301102855 | 1.05E-80 | 101107420 | LOC101107420 | -4.79433213 | 2.29E-53 |
| 101108392 | CPLX2 | 5.281632991 | 1.09E-05 | 101113672 | LOC101113672 | 4.785313799 | 6.64E-31 |
| 101111766 | ATP2A1 | 5.232002223 | 2.54E-86 | 114116368 | LOC114116368 | -4.763107413 | 3.30E-04 |
| 101111132 | TNNC1 | 5.079999129 | 5.08E-05 | 105610137 | LOC105610137 | 4.740316063 | 6.64E-04 |
| 101108520 | LOC101108520 | 5.079999129 | 5.08E-05 | 101111554 | ACTC1 | -4.739423788 | 2.76E-07 |
| 101120204 | LOC101120204 | 5.07784555 | 0 | 114112432 | LOC114112432 | 4.728548502 | 7.05E-04 |
| 114113983 | LOC114113983 | 5.05638363 | 2.68E-258 | 101114332 | GRID1 | 4.654610107 | 4.88E-28 |
| 101113335 | LOC101113335 | -5.049283888 | 9.28E-08 | 101113062 | ASIC3 | -4.636330295 | 8.36E-07 |
| 101122145 | MROH7 | 5.03419544 | 1.60E-33 | 101122163 | LOC101122163 | -4.511382203 | 0.001220777 |
| 442995 | LOC442995 | 4.938908457 | 1.29E-23 | 114112282 | LOC114112282 | 4.479744732 | 0.023160209 |
| 101122163 | LOC101122163 | 4.915923204 | 1.55E-04 | 100303604 | KRT17 | -4.466405294 | 0.001510618 |
| 101104467 | TNNT3 | 4.902729277 | 4.79E-120 | 101113097 | DSCAML1 | -4.466405294 | 4.42E-06 |
| 101112298 | LOC101112298 | -4.863687822 | 7.49E-04 | 101108975 | DPYSL4 | -4.466405294 | 0.001510618 |
| 105608827 | LOC105608827 | 4.814358482 | 3.29E-29 | 101115395 | PGLYRP1 | -4.466405294 | 0.001510618 |
| 114109097 | LOC114109097 | 4.783985733 | 3.44E-04 | 114116116 | LOC114116116 | -4.459777742 | 4.69E-06 |
| 101110777 | LOC101110777 | 4.758071034 | 3.99E-04 | 101111035 | LOC101111035 | -4.444201236 | 0 |
| 101117301 | C25H10orf71 | 4.758071034 | 3.99E-04 | 101112298 | LOC101112298 | -4.341530033 | 1.33E-05 |
| 101119458 | GTSF1 | -4.723055655 | 3.73E-17 | 101103742 | PRRX1 | -4.340874412 | 0.002656657 |
| 443471 | MYH2 | 4.50115509 | 1.25E-11 | 101115178 | WDR49 | 4.288482208 | 0.004971864 |
| 101106651 | SFTPD | -4.489856479 | 5.55E-19 | 101113861 | MED4 | -4.264383731 | 2.34E-25 |
| 101113622 | SCN4B | 4.458510753 | 0.001925341 | 114108618 | LOC114108618 | 4.262803932 | 0 |
| 101114720 | LOC101114720 | -4.394575976 | 6.39E-239 | 114108784 | LOC114108784 | -4.243952274 | 5.44E-22 |

| Table S4. Top 50 DEGs in liver at different temperatures in Altay and Hu lambs. | | | | | | | |
| --- | --- | --- | --- | --- | --- | --- | --- |
| A-liver^c^-H-liver^c^ | | | | A-liver^w^-H-liver^w^ | | | |
| Gene ID | Gene Symbol | log2FC | Qvalue | Gene ID | Gene Symbol | log2FC | Qvalue |
| 101112841 | CKM | 10.53405387 | 1.50E-142 | 114112432 | LOC114112432 | 9.380861125 | 5.34E-66 |
| 101118481 | LOC101118481 | 10.44686379 | 0 | 114116451 | LOC114116451 | 8.791888052 | 1.94E-47 |
| 101107662 | ACTA1 | 10.00075349 | 2.00E-213 | 114108993 | LOC114108993 | -8.370465446 | 6.14E-31 |
| 442994 | MYH1 | 9.010687584 | 8.31E-62 | 114112166 | LOC114112166 | 7.974723935 | 1.16E-29 |
| 100127216 | TNNC2 | 8.907160694 | 2.68E-58 | 101122145 | MROH7 | -7.765232915 | 7.82E-170 |
| 114117594 | LOC114117594 | 8.751069119 | 2.30E-53 | 114115573 | LOC114115573 | -7.530912493 | 3.09E-233 |
| 114112267 | LOC114112267 | -8.366159974 | 2.48E-97 | 101111827 | LOC101111827 | 7.438860563 | 1.20E-21 |
| 100270715 | MYLPF | 8.050629401 | 1.12E-70 | 114113605 | LOC114113605 | 6.995143988 | 1.33E-16 |
| 101105132 | LOC101105132 | 7.826263013 | 3.23E-31 | 114115271 | LOC114115271 | 6.736289579 | 3.35E-14 |
| 101109898 | EEF1A2 | 7.798757101 | 1.02E-30 | 114113983 | LOC114113983 | -6.52721487 | 0 |
| 443220 | GFAP | 7.77351981 | 6.13E-60 | 101111100 | CXCL13 | -6.465209513 | 1.19E-56 |
| 780509 | MB | 7.752146159 | 3.55E-59 | 114113045 | LOC114113045 | 6.428529889 | 2.22E-68 |
| 101112304 | KRT6A | 7.695045752 | 6.84E-29 | 101105132 | LOC101105132 | -6.285269543 | 4.51E-09 |
| 114112309 | LOC114112309 | 7.650855345 | 1.06E-98 | 101119157 | MMP12 | -5.975824672 | 1.56E-07 |
| 114115271 | LOC114115271 | -7.635420214 | 3.54E-18 | 101107420 | LOC101107420 | -5.866346196 | 4.17E-63 |
| 100303604 | KRT17 | 7.544037964 | 2.08E-26 | 105603393 | LOC105603393 | 5.779463523 | 6.45E-08 |
| 101101869 | MYL1 | 7.397551875 | 3.44E-24 | 101108719 | PAX5 | -5.734816573 | 1.62E-06 |
| 105613035 | TCHH | 7.246771923 | 4.37E-22 | 101114720 | LOC101114720 | 5.684616978 | 4.06E-214 |
| 101121072 | PLP1 | 7.085228062 | 1.98E-39 | 101120468 | CCL27 | -5.663574491 | 3.02E-06 |
| 443527 | PRD-SPRRII | 6.88769083 | 9.47E-18 | 101104704 | UPK1B | -5.560787173 | 7.39E-11 |
| 101114569 | COL24A1 | -6.723333968 | 1.40E-10 | 101113335 | LOC101113335 | 5.531969968 | 8.48E-07 |
| 114110327 | LOC114110327 | 6.53138678 | 0 | 100462743 | DUOX2 | -5.523312468 | 9.62E-06 |
| 101108767 | CACNA1S | 6.408522993 | 3.34E-13 | 114109097 | LOC114109097 | -5.338887897 | 3.80E-05 |
| 101117175 | KRT36 | 6.364128874 | 7.69E-13 | 114111248 | LOC114111248 | 5.312124702 | 1.35E-23 |
| 101113800 | MOBP | 6.197018888 | 1.44E-11 | 101108520 | LOC101108520 | -5.082739876 | 2.00E-04 |
| 101109031 | IGFN1 | 6.007985063 | 2.86E-10 | 114117593 | LOC114117593 | -5.064070665 | 2.23E-04 |
| 101105314 | LOC101105314 | 5.918755903 | 2.45E-73 | 100101227 | WT1 | -4.975824672 | 3.71E-04 |
| 101104853 | SLN | 5.918717725 | 1.05E-09 | 101122163 | LOC101122163 | -4.905324364 | 5.45E-04 |
| 101105997 | LOC101105997 | 5.790393628 | 5.96E-09 | 101119401 | COL22A1 | -4.734816573 | 2.58E-06 |
| 114108702 | LOC114108702 | 5.720976953 | 1.45E-08 | 114115623 | LOC114115623 | 4.725017527 | 1.65E-15 |
| 105608827 | LOC105608827 | 5.716192128 | 7.26E-27 | 101110048 | RFX8 | 4.724615046 | 1.71E-20 |
| 114108648 | ANKS1B | 5.686056968 | 2.23E-08 | 101103362 | PNLIPRP3 | 4.724615046 | 3.70E-04 |
| 100196904 | MYL2 | 5.612056387 | 5.39E-08 | 101113487 | LOC101113487 | -4.597313049 | 9.06E-06 |
| 114111248 | LOC114111248 | 5.53564781 | 1.67E-68 | 101107140 | BNC1 | -4.597313049 | 9.06E-06 |
| 101104467 | TNNT3 | 5.508787116 | 2.05E-109 | 114110308 | LOC114110308 | -4.522293043 | 0 |
| 101113861 | MED4 | 5.247333442 | 2.31E-64 | 114113815 | LOC114113815 | -4.475449816 | 0.004006178 |
| 101119079 | LOC101119079 | 5.222109869 | 1.04E-11 | 100048994 | CYP11A1 | -4.471782167 | 3.72E-14 |
| 101111766 | ATP2A1 | 5.200630141 | 7.53E-77 | 101101949 | VGLL3 | -4.445309956 | 0.004520401 |
| 114113045 | LOC114113045 | 5.183717061 | 8.24E-31 | 101108767 | CACNA1S | -4.445309956 | 3.21E-05 |
| 443471 | MYH2 | 5.107212929 | 8.06E-11 | 101106651 | SFTPD | 4.37799401 | 6.76E-16 |
| 114115658 | LOC114115658 | 4.960804795 | 0.007008677 | 100431098 | SCGB1A1 | -4.362847795 | 6.06E-05 |
| 101103751 | ATP1A3 | 4.949091374 | 3.05E-05 | 101102938 | TMEM40 | -4.362847795 | 6.06E-05 |
| 101117253 | CGAS | 4.944877365 | 3.15E-05 | 101122151 | ITLN2 | -4.317153604 | 8.46E-05 |
| 101115178 | WDR49 | -4.805796128 | 0.002225723 | 101123372 | LOC101123372 | -4.302569784 | 1.49E-90 |
| 101102105 | LOC101102105 | 4.756446296 | 1.23E-04 | 101119177 | GP2 | -4.275384954 | 0.00851827 |
| 101111132 | TNNC1 | 4.686056968 | 1.95E-04 | 101118751 | LOC101118751 | -4.275384954 | 0.00851827 |
| 101107420 | LOC101107420 | -4.635871126 | 0.004535817 | 101116368 | LOC101116368 | -4.275384954 | 0.00851827 |
| 442995 | LOC442995 | 4.544966296 | 1.73E-20 | 105603087 | LOC105603087 | -4.232028585 | 1.94E-15 |
| 101122163 | LOC101122163 | 4.521981043 | 5.29E-04 | 101116587 | ALOX15 | -4.222917534 | 2.65E-26 |
| 101111178 | LOC101111178 | 4.505725152 | 5.79E-04 | 101112836 | DISP3 | 4.184046664 | 0.004995557 |
